# Intradiscal Condoliase Injection Therapy for Recurrent Lumbar Disc Herniation: Case Series and Literature Review

**DOI:** 10.3390/medicina59091561

**Published:** 2023-08-28

**Authors:** Hiroki Fukui, Naosuke Kamei, Yasushi Fujiwara, Toshio Nakamae, Ryo Ohta, Shinji Kotaka, Nobuo Adachi

**Affiliations:** 1 Orthopedics and Micro-Surgical Spine Center, Hiroshima City North Medical Center Asa Citizens Hospital, Hiroshima 731-0293, Japan; ys.fujiwara@nifty.com (Y.F.); xxxhr307@yahoo.co.jp (R.O.); kotaka_shinji1216@yahoo.co.jp (S.K.); 2 Department of Orthopaedic Surgery, Graduate School of Biomedical and Health Sciences, Hiroshima University, Hiroshima 734-8551, Japan; nakamei@hiroshima-u.ac.jp (N.K.); toshinakamae623813@yahoo.co.jp (T.N.); nadachi@hiroshima-u.ac.jp (N.A.)

**Keywords:** intervertebral disc, herniation, recurrent, condoliase, chondroitinase ABC

## Abstract

*Background and objectives*: Although chemonucleolysis with condoliase for lumbar disc herniation (LDH) has become common, few reports have described its application in the treatment of recurrent LDH. Therefore, this study aimed to evaluate the safety and efficacy of condoliase treatment in six patients with recurrent LDH and review the available literature on condoliase treatment for LDH. *Materials and Methods*: Six patients (four men and two women; mean age, 64.7 years) with recurrent LDH who were treated with condoliase at our hospital between 2019 and 2022 were included. The clinical records and images of the patients were retrospectively evaluated. In addition, the available English literature on condoliase treatment for LDH was retrieved and reviewed. *Results*: Among the six patients included in the study, three showed >50% improvement in leg pain after treatment, which is a lower efficacy rate than that in previous reports. In addition, two patients required surgery after treatment, which is a higher rate than that in previous reports. The mean intervertebral disc height significantly decreased from 8.4 mm before treatment to 6.9 mm after treatment, consistent with the results of previous studies. None of the cases showed Modic type I changes on magnetic resonance imaging. *Conclusions*: Although the efficacy of condoliase treatment for recurrent LDH may be lower than that for primary LDH, this treatment was found to be safe and applicable for recurrent LDH.

## 1. Introduction

Despite the generally satisfactory outcomes of discectomy for lumbar disc herniation (LDH), recurrence remains a persistent issue. Conservative therapy is recommended for patients with recurrent LDH. However, revision surgery is often required when conservative measures are ineffective. A longitudinal observational study of 34,639 surgeries revealed that 2.1% of the patients required revision surgery [1,2]. Another study of patients with severe and persistent LDH-associated sciatic pain conducted over a 13-year follow-up period reported that 8% of the patients who were treated surgically required revision surgery at the same level [3]. However, the technical difficulties associated with revision surgery owing to the presence of epidural scars remain a concern [4,5,6,7,8].

A new chemonucleolytic therapy using condoliase was recently developed for LDH treatment [9,10]. Condoliase, also known as chondroitinase ABC, is a chondroitin sulfate ABC endolyase that received approval for LDH treatment in Japan in 2018. LDH treatment using condoliase received insurance coverage in 2019 and has subsequently become a frequently used technique in general practice. Condoliase injections into the disc selectively degrade chondroitin sulfate, a mucopolysaccharide component of the nucleus pulposus; this results in a reduced herniated disc size, which often negates the need for surgery. Although numerous reports have described the efficacy of condoliase injection therapy, very few studies have described its application in the treatment of recurrent LDH [11,12,13]. Therefore, we presented a case series of six patients who were treated with condoliase for recurrent LDH. In addition, we reviewed previous studies on condoliase treatment for LDH and compared their findings with the results of our study.

## 2. Materials and Methods

### 2.1. Case Series

#### 2.1.1. Participants

In total, 170 patients underwent condoliase treatment (HERNICORE^®^ 1.25 units in 1 mL for intradiscal injection; Kaken Pharmaceutical, Tokyo, Japan) at our hospital between October 2019 and May 2022. Of these, six patients were included in this study because they had previously undergone lumbar discectomy at our hospital and had recurrent LDH at the same level. Patients were informed about condoliase and surgical treatments and those who accepted condoliase treatment were included in the study.

#### 2.1.2. Condoliase Treatment

**Figure 1:**
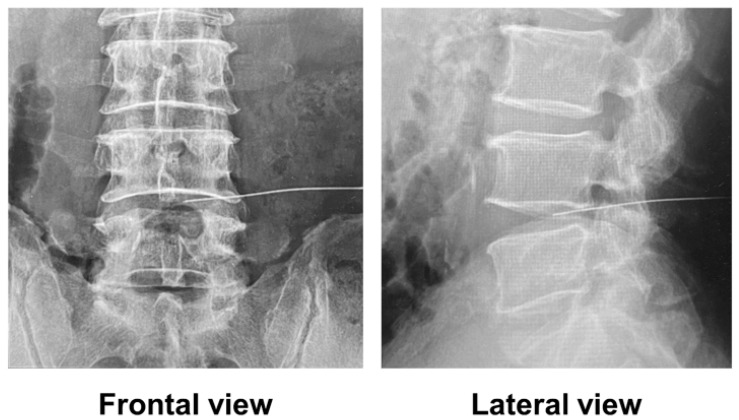
Fluoroscopic images immediately before condoliase injection.

Treatment was performed in accordance with the guidelines for proper use provided by the pharmaceutical company (Kaken Pharmaceutical, Tokyo, Japan). In accordance with the protocol, the patients were admitted to the hospital, and the procedure was conducted in a fluoroscopy room. The patients were placed in the prone position, and fluoroscopic frontal and lateral views of the lumbar spine confirmed that the needle tip was approximately at the center of the intervertebral disc (Figure 1). Afterward, the needle was inserted into the disc from the herniated side in an oblique view of the lumbar spine, and condoliase was injected. Patients were observed at rest for at least 3 h after treatment.

#### 2.1.3. Evaluations

The visual analog scale (VAS) scores for low back pain and leg pain and the Japanese Orthopaedic Association (JOA) scores were assessed before and 1, 3, and 6 months after condoliase treatment. Intervertebral disc degeneration was evaluated using radiography and magnetic resonance imaging (MRI). Before and 6 months after treatment, intervertebral disc height was examined using radiography, and the Pfirrmann criteria were used to evaluate disc degeneration on MRI [14]. The Pfirrmann grades were evaluated by two spine surgeons (H.F. and N.K.), and in cases with disagreements, the worst grade was selected. In addition, vertebral endplates were evaluated using the Modic classification on MRI [15]. LDH type and changes in herniation size were evaluated using MRI [16].

#### 2.1.4. Statistical Analysis

Statistical analysis of the changes in VAS and JOA scores before and after treatment was performed using one-way repeated-measures analysis of variance followed by Dunnett’s post hoc test. Changes in intervertebral disc height before and after treatment were analyzed using a paired *t*-test.

### 2.2. Literature Review

**Figure 2:**
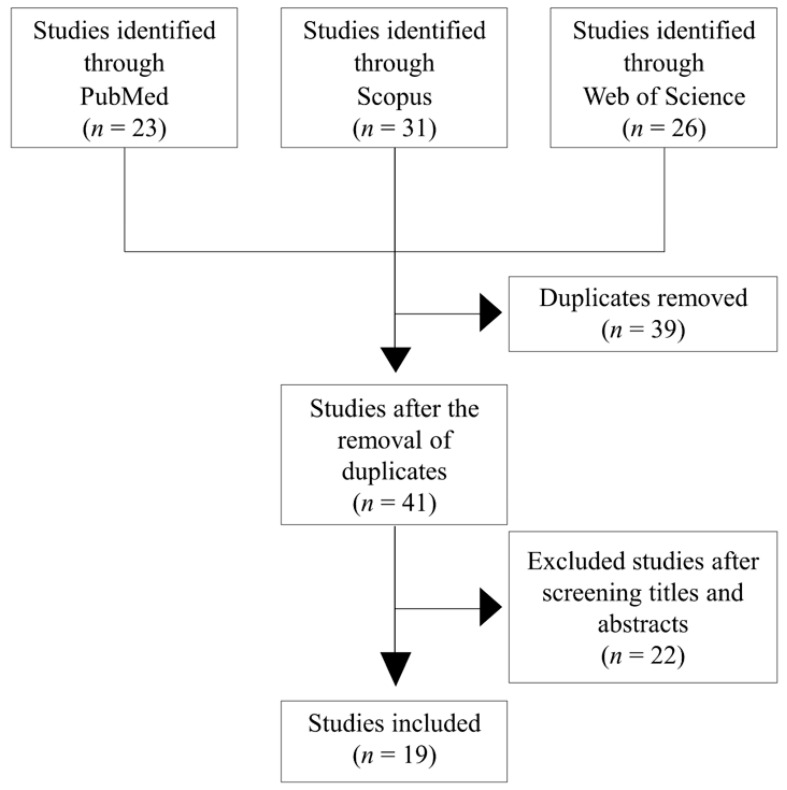
Flow diagram of the study search and selection process.

We conducted a comprehensive review of existing original articles on the clinical application of condoliase for the treatment of LDH. Previous studies were retrieved from three major academic databases (PubMed, Scopus, and Web of Science) using the appropriate search term combinations, including “condoliase” OR “chondroitinase ABC” OR “chondroitin sulfate ABC endolyase” AND “lumbar disc herniation.” Basic research and review articles were excluded. Ultimately, 19 articles were selected, including three randomized controlled trials [9,10,17], 14 case series [12,13,18,19,20,21,22,23,24,25,26,27,28,29], and two case reports [11,30] (Figure 2). Patient demographics were examined, including age, sex, LDH level, Pfirrmann grade, efficacy rate, and post-treatment surgical rate in patients with LDH treated with 1.25 units of condoliase. Case reports were excluded and individually reviewed.

## 3. Results

Data from our case series of six patients (four men and two women) are presented in Table 1.

**Figure 3:**
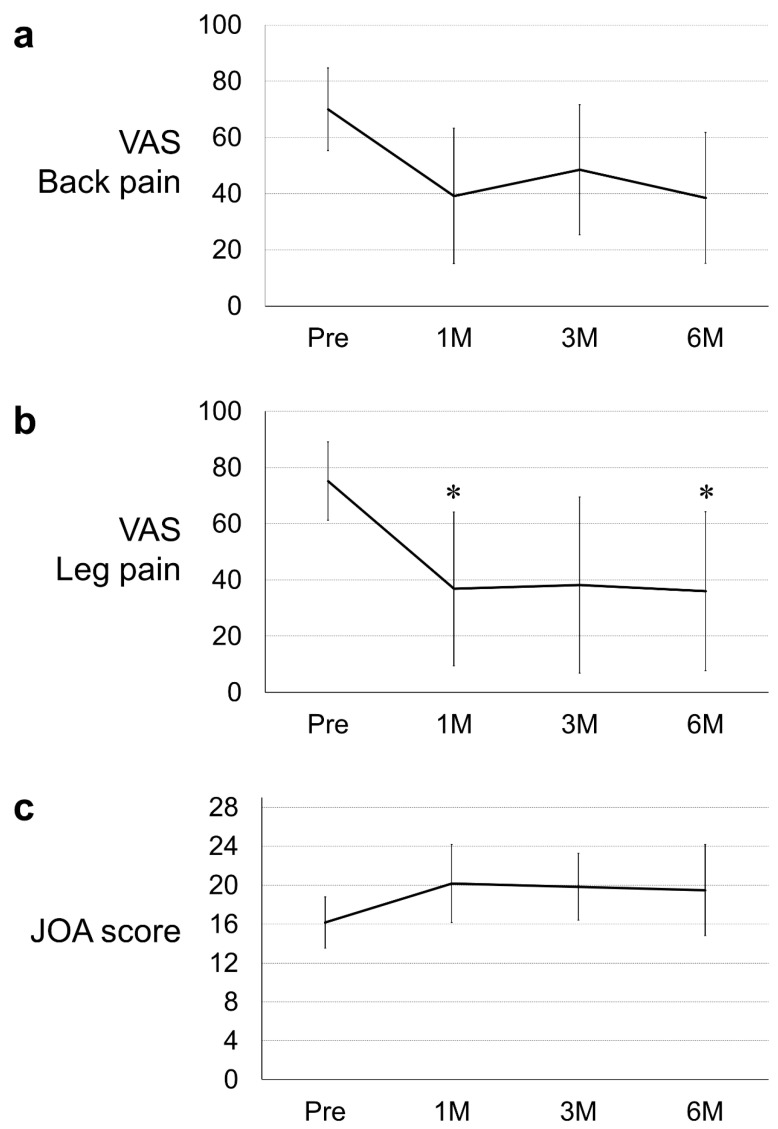
Line graph of visual analog scale (VAS) score for (**a**) low back and (**b**) leg pain, and (**c**) Japanese Orthopaedic Association (JOA) score before and 1, 3, and 6 months (M) after treatment. * Significantly better score than the pretreatment score, *p* < 0.050.

The mean age of the patients was 64.7 years (range: 48–82 years); two patients experienced herniated discs at L4-5 and four patients at L5-S1. The mean time to LDH recurrence was 21.5 months (range: 6–48 months) after initial surgery, and the mean time from LDH recurrence to condoliase treatment was 20.8 weeks (range: 1–52 weeks). Each of the six patients received a different oral analgesic therapy, including celecoxib, loxoprofen, acetaminophen, pregabalin, mirogabalin, and neurotropin, before treatment. There was no change in post-treatment oral therapy. In all the patients, the leg pain was radicular pain consistent with an area innervated by a nerve root compressed by a herniated disc. The mean VAS score for low back pain decreased from 70.0 before treatment to 39.2, 48.5, and 38.5 at 1, 3, and 6 months after treatment, respectively. However, no significant differences were observed between the pre- and post-treatment scores (1 month 95% confidential interval (CI), 20.7–57.6; *p* = 0.058, 3 months 95% CI, 30.1–66.9; *p* = 0.233, and 6 months 95% CI, 20.1–56.9; *p* = 0.052) (Figure 3a). In contrast, the mean VAS score for leg pain decreased from 75.2 before treatment to 36.8, 38.2, and 36.2 at 1, 3, and 6 months after treatment, respectively, with significant differences observed at 1 month (95% CI, 14.6–59.0; *p* = 0.049) and 6 months (95% CI, 13.8–58.2; *p* = 0.044) after treatment (Figure 3b). Six months after treatment, the VAS score for leg pain decreased to ˂50% of the pretreatment levels in three of the six patients. The mean JOA score increased from 16.2 before treatment to 20.2, 19.8, and 19.5 at 1, 3, and 6 months after treatment, respectively. However, the pre- and post-treatment scores showed no significant differences (1 month 95% CI, 17.0–23.4; *p* = 0.191; 3 months 95% CI, 16.6–23.0; *p* = 0.247; and 6 months 95% CI, 16.3–22.7; *p* = 0.315) [Figure 3c].

**Figure 4:**
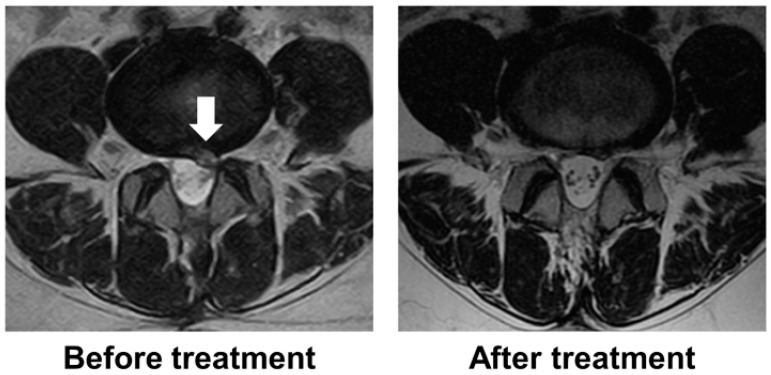
Representative magnetic resonance images before and 3 months after the treatment of a patient with herniated disc resolved by condoliase treatment. A white arrow indicates a herniated disc.

The mean intervertebral disc height significantly decreased from 8.4 mm before treatment to 6.9 mm after treatment (95% CI, 0.990–1.877; *p* < 0.001). MRI evaluation showed that five of the six patients had T2 high signal intensity in the herniated disc before treatment. Recurrent disc herniation types were protrusion in two patients and subligamentous extrusion in four patients. All herniated discs were located laterally in the spinal canal and compressed nerve roots. There were no massive herniated discs compressing the cauda equina. Before treatment, the Pfirrmann grades were III in two patients, IV in two patients, and V in two patients. After treatment, the Pfirrmann grade changed from III to IV in one patient. Disc herniation resolved in two patients after condoliase treatment (Figure 4) but persisted in four patients. Of the four patients with residual herniation, two showed a reduction in the size of the herniation, and two showed no change in size. The two patients who showed no change in LDH size required revision surgery at 7 and 12 months post-treatment.

Data from previous reports on patients treated with condoliase for LDH are summarized in Table 2.

The number of patients included in each study ranged from 26 to 228, and their mean age was 21.1–53.1 years. There were more women (20.8–42.6%) than men in all of the reports. Most LDH locations were at the L4-5 or L5-S1 level. Prior to treatment, most patients had Pfirrmann grades II-IV, with grade III being the most common. The most common type of LDH was subligamentous extrusion, with an incidence rate of 43.8–72.3%. Efficacy was primarily defined as ≥50% improvement in leg pain, with efficacy rates of 61.8–85.4%. The percentage of patients who required surgery after treatment was 5.8–13.1%. The mean decrease in disc height after treatment was 10.1–20.0%, and the percentage of patients with a change in Pfirrmann grade as an indicator of disc degeneration after treatment was 38.6–61.5%.

Of the aforementioned studies, three included cases of postoperative LDH recurrence. One study reported six recurrent cases but did not mention the outcomes for these patients [21]. Another study reported that the VAS score for leg pain did not improve by >20 mm in any of the three recurrent cases [13]. In contrast, another study reported that of the eight patients with a history of discectomy at the same level as the LDH, six (75.0%) were responders with a ≥50% improvement in the VAS score for leg pain [12]. In addition, one case report described a patient with postoperative LDH recurrence whose LDH shrank after treatment and whose VAS score for leg pain improved from 90 mm before treatment to 30 mm 3 months after treatment [11].

## 4. Discussion

In the present study, we evaluated six patients with postoperative recurrent LDH using VAS and JOA scores, radiographs, and MRI scans before and after condoliase treatment. Our results showed that leg pain improved to ˂50% of the pretreatment levels in three patients, whereas surgery was required in two of the remaining three patients.

Our case series showed a higher mean age and a higher proportion of Pfirrmann grade V cases than did previous reports; however, it had a sex ratio and distribution of LDH locations similar to those of other studies. Leg pain improved by more than 50% after treatment in three of the six patients included in the study, which is a lower efficacy rate than that in previous reports. In addition, surgery was required after treatment in 33.3% of our patients (two of six), which is a higher rate than that reported in previous studies. Although most previous studies did not observe an association between age and efficacy, only one study showed significantly higher efficacy in younger patients in univariable analysis, with no significant association in multivariable analysis [19]. This finding suggests that age is not strongly associated with efficacy. In addition, no previous reports have shown a significant association between efficacy and Pfirrmann grade. Studies describing the efficacy of condoliase treatment for postoperative recurrence of LDH have yielded mixed results [11,12,13]. Although the degree of efficacy is not clear owing to the small number of cases, including in the cases included in our study, the treatment is clearly effective in some cases.

A previous randomized controlled trial reported that the most common adverse events in patients treated with condoliase compared with placebo controls were back pain (36.6% in the condoliase group and 33.3% in the placebo group), Modic type 1 changes (26.8% in the condoliase group and 17.3% in the placebo group), and disc height reductions of 30% or more (8.5% in the condoliase group and 0% in the placebo group) [9]. In our case series, we did not observe any of these adverse events or any other adverse events. No specific adverse events were reported in previous studies regarding the treatment of postoperative LDH recurrence. The safety of condoliase treatment for postoperative LDH recurrence appears to be acceptable. Moreover, condoliase treatment is more cost-effective than surgical treatment and can be a cost-effective alternative to nonsurgical, conservative therapy [27]. Although condoliase treatment for postoperative LDH recurrence may be less effective than treatment for primary LDH, it is minimally invasive, safe, and cost-effective. Therefore, it may be considered the first modality of choice.

A limitation of our study is that it did not include comparisons with treatment for primary LDH; in addition, the small number of patients did not allow us to determine the differences in the efficacy of treatment for primary LDH. Future comparative studies with larger numbers of patients are warranted.

## 5. Conclusions

Among six patients with postoperative recurrent LDH, three showed >50% improvement in leg pain after condoliase treatment, whereas two required additional surgery. The treatment was safe and had no adverse effects. Condoliase treatment can also be applicable to recurrent LDH.

## Figures and Tables

**Table 1:** Summary of the findings for the six patients in our case series.

		Patient 1	Patient 2	Patient 3	Patient 4	Patient 5	Patient 6	*p*-Value
Age (years)		82	61	51	48	76	70	
Sex		Male	Male	Male	Female	Male	Female	
Location		L5-S	L5-S	L5-S	L4-5	L4-5	L5-S	
Time to recurrence (month)		24	15	6	12	24	48	
Time to treatment (week)		16	52	20	1	8	28	
Follow-up time (month)		12	24	7	12	12	12	
VAS score forback pain (mm)	Pre	56	71	86	85	72	50	
	1 M	22	25	81	21	55	31	0.058
	3 M	67	54	75	23	54	18	0.233
	6 M	48	52	73	19	28	11	0.052
VAS score forleg pain (mm)	Pre	78	74	84	85	82	48	
	1 M	31	26	81	11	58	14	0.049
	3 M	35	55	81	0	53	5	0.059
	6 M	50	52	75	0	29	10	0.044
JOS score	Pre	20	18	15	14	13	17	
	1 M	17	24	16	20	18	26	0.191
	3 M	19	24	16	19	17	24	0.247
	6 M	21	23	20	18	11	24	0.315
Disc height (mm)	Pre	8.7	9.6	5.5	7.6	9.7	9.0	<0.001
	6 M	6.9	8.1	3.7	6.4	9.0	7.4	
T2 signal intensity of disc herniation		High	High	High	High	Low	High	
Pfirrmann grade	Pre	IV	III	V	III	IV	V	
	6 M	IV	IV	V	III	IV	V	
Type of disc herniation		PT	SL	SL	PT	PT	SL	
Size of disc herniation		No change	Reduced	No change	Disappeared	Reduced	Disappeared	
Time to reoperation (month)		12	-	7	-	-	-	

M, month; Pre, pretreatment; PT, protrusion; SL, subligamentous.

**Table 2:** Summary of previously reported cases of condoliase treatment for lumbar disc herniation.

Number of patients		26–228
Mean age (years)		21.1–53.1
Sex (percentage of women)		20.8–42.6
Location (%)	L1–2	0–1.9
	L2–3	0–6.1
	L3–4	0–11.9
	L4–5	40.6–73.5
	L5–S1	26.5–48.3
Pfirrmann grade (%)	I	0–1.2
	II	3.1–38.2
	III	32.7–80.8
	IV	2.9–53.8
	V	0–4.4
Type of LDH (%)	Protrusion	0–16.9
	Subligamentous	43.8–72.3
	Transligamentous	0–56.3
Efficacy rate (%)		61.8–85.4
Surgery rate (%)		5.8–13.1
Mean decrease in disc height (%)		10.1–20.0
Percentage of cases showing a change in Pfirrmann grade (%)		38.6–61.5

## Data Availability

The data presented in this study are available on request from the corresponding author.
